# Modeling complexity: cognitive constraints and computational model-building in integrative systems biology

**DOI:** 10.1007/s40656-017-0183-9

**Published:** 2018-01-08

**Authors:** Miles MacLeod, Nancy J. Nersessian

**Affiliations:** 1 Department of Philosophy, University of Twente, Drienerlolaan 5, 7522 NB Enschede, The Netherlands; 2 Department of Psychology, Harvard University, 33 Kirkland St., Cambridge, MA 02138 USA

**Keywords:** Scientific cognition, Simulation, Systems biology, Mental modeling, Complexity

## Abstract

Modern integrative systems biology defines itself by the complexity of the problems it takes on through computational modeling and simulation. However in integrative systems biology computers do not solve problems alone. Problem solving depends as ever on human cognitive resources. Current philosophical accounts hint at their importance, but it remains to be understood what roles human cognition plays in computational modeling. In this paper we focus on practices through which modelers in systems biology use computational simulation and other tools to handle the cognitive complexity of their modeling problems so as to be able to make significant contributions to understanding, intervening in, and controlling complex biological systems. We thus show how cognition, especially processes of simulative mental modeling, is implicated centrally in processes of model-building. At the same time we suggest how the representational choices of what to model in systems biology are limited or constrained as a result. Such constraints help us both understand and rationalize the restricted form that problem solving takes in the field and why its results do not always measure up to expectations.

## Introduction

Modern computational science is complex: cognitively, technologically, and collaboratively. A prime example is the field of integrative systems biology (ISB). This field aims to understand, intervene on, and control biological systems comprising integrated, interacting, complex networks of genes, proteins, and biochemical reactions. In the field of ISB those who do computational modeling (as opposed to data mining) tend to use “computational model(ing)” and “simulation” interchangeably because the purpose of building a computational model in ISB is to run simulations of actual and counterfactual system dynamics. Model simulation is the process through which models both are built and are tested. Solutions to the problems the field poses create an essential *interdependence* among the participating fields: computational sciences, engineering sciences, and biological sciences. The nature of the problems posed in integrative systems biology requires both specialization and collaboration. Although there are some on-going attempts to develop hybrid modeler-experimentalists, in principle, modelers (mainly engineers, physicists, and applied mathematicians) and experimentalists (mainly molecular biologists and biochemists) have a symbiotic relationship. One bioscientist we interviewed characterized the situation succinctly:

Number one, team science is the only way it’s gonna work these days. It’s really gonna get hard to write a single investigator RO1 these days and expect to get funded because everyone is now realizing the interconnectedness of everything. And for me to be able to sit here and think that I can have all the expertise in my tiny little brain to do everything with all these approaches that I don’t understand at all is ridiculous…that’s not how it’s (bimodal) ever really gonna work… at the PI level because you’re gonna be much more on one side than the other. So you need the other half of your [bioscientist] brain to be in another person, G4 [a modeler]. For me, to be [in] G4.^1^

However, with little knowledge of one another’s methods, concepts, technologies, and epistemic values, at the present time symbiosis is more a desideratum than a reality. Although the field is young, developing, and diverse, the challenges of collaboration that were detailed in our interviews of both modelers and experimentalists are widespread, which we see when presenting our claims to wider audiences of systems biologists. In the absence of effective collaborations, the lack of biological knowledge and access to sufficient data for building models increases the inherent cognitive complexity of the task for modelers. Further, unlike the situations of physics-based and climate science modeling that predominates in the philosophical literature on computational modeling and simulation (see for instance Winsberg 2010; Parker 2010; Lenhard 2006, 2007; Humphreys 2004) systems biology lacks well established domain theories which can provide a platform of representational resources and methods for developing reliable simulation models (see MacLeod and Nersessian 2013a). The modelers we have studied bring with them tools and tricks from engineering, but all aspects of the process of modeling complex biological systems are open to decision, including:

- Representations of biochemical interactions to use (Michaelis–Menten, power laws, etc.)
- Data sets and databases to use.
- Pathway elements to include or exclude.
- Parameter estimation techniques, parameter-fixing algorithms, algorithm development.

We have conducted a 5-year ethnographic investigation of modeling practices in two pioneering integrative systems biology laboratories in which the biosystems modelers mostly had engineering backgrounds.^2^ Lab G conducts only computational modeling. Lab C conducts computational modeling and modelers also conduct their own bench-top experiments in service of model-building. Lab C researchers have the ability to incorporate experiment into their practices which leads to hybrid experiment-modeling strategies (see MacLeod and Nersessian 2013b). We label these researchers “bi-modal”. In this paper however we restrict ourselves to the practices mostly used in Lab G. Although there is a range of ways in which systems biology is practiced, dedicated computational labs which collaborate with external experimentalists predominate. Our primary goals in this paper are (1) to help understand how these “uni-modal” computational modelers are able to handle the complexity of their modeling problems cognitively so as to build at least partially accurate models, and in turn make sometimes profound contributions to the understanding of the systems they are modeling; and (2) to understand, to the extent possible given our data, the ways in which cognitive capacities and constraints play a role in the representations they build and methodological choices they make. These choices might initially appear ineffective given the epistemological goals of the field, but can be rationalized nonetheless on cognitive grounds.

In this respect our paper both builds upon and diverges from the traditional manner through which cognitive practices in science have been studied in philosophy of science and elsewhere.^3^ Various philosophers for instance have studied the cognitive affordances of particular model-building and other strategies for building representations of phenomena. Wimsatt for instance has leveraged Levin’s original discussion of modeling strategies for simplifying and idealizing complex biological systems (in population biology), and Simon’s work on problem-solving, into a cognitive theory of the affordances and biases of common reductionistic heuristics in scientific practice (see Wimsatt 2007; see also Bechtel and Richardson’s discussion of the role of decomposition and localization heuristics in handling complex systems). Wimsatt’s work has precipitated a line of discussion on the role that false models, idealized models and robustness analysis play in scientific discovery processes (e.g. Weisberg 2006). This research helps both understand and rationalize the steps modelers typically employ to break down complex systems and recover information about these systems from their models given cognitive and other constraints.

When trying to account for the methodological choices and abilities of systems biologists to derive information from complex biochemical systems we want to illustrate in this paper an important role for a cognitive analysis that goes deeper than the analysis of heuristics, to consider actual cognitive mechanisms and processes modelers rely upon to process information through the use of their models. Part of the need for this as we will see is that many of the inferences and decisions modelers make about how to structure and improve their models are dependent on the ways in which computational simulations are used to help augment and direct their own cognitive capacities. As the title of Humphreys (2004) book advertises, computational simulation provides a novel way of “extending ourselves;” that is, it provides fundamentally new ways of doing science through extending human cognitive capacities. But, this and other analyses mostly hint at the nature of these capacities without providing a precise account of the cognitive functions and factors which underlie them.

To develop this account, we need to draw from research in the cognitive sciences, particularly in the area of mental modeling, mental simulation, model-based reasoning and distributed cognition. On our account, just as the microscope and telescope extended the human capacity for “seeing,” computational simulation has extended the human cognitive capacity for reasoning via mentally simulating dynamical phenomena (“simulative model-based reasoning”^4^). The account we have been developing moves away from cognitive models that focus exclusively on individual cognitive processes and draws from the cognitive frameworks of distributed cognition and of simulative mental modeling to cast the modeler and model as constituting a “coupled cognitive system” through which model-based inferences are made (Nersessian 2002, 2008; Chandrasekharan et al. 2012; Chandrasekharan and Nersessian 2015; Chandrasekharan and Nersessian, forthcoming). Accounts of distributed cognition in science have been proposed by others in the philosophical literature (see, e.g., Giere 2002), but these accounts focus on the collective or socially distributed nature of cognition, rather than the distribution among instruments, artifacts and technologies, and a human agent. Despite the interdisciplinary nature of systems biology, the process of model-building is mostly the responsibility of the individual modeler rather than well-coordinated collective processes between modelers and experimentalists. This is largely because the computational model is a “black box” to most experimentalists.

As such in this paper we aim to illustrate the value of richer cognitive accounts for explaining problem-solving processes and methodological choices in a computational field such as ISB. To this end in Sect. 2 we begin by considering the complex nature of the problem solving tasks which confront modelers in systems biology, and the kinds of inferences they are able to make in order to improve their models despite this complexity. In Sect. 3 we discuss the potential importance of distributed cognition and of mental modeling as the cognitive mechanisms through which modelers produce these inferences. In Sect. 4 we show the potential for such a cognitive account to help explain and rationalize various aspects of methodological choice in the field. The size and scale of networks being modeled by systems biologists seem too small for obtaining central goals in the field, particularly with respect to prediction (Voit et al. 2012a, b). We will suggest that the size and scale of network which can be represented is constrained by limits on the operational effectiveness of those cognitive practices modelers rely on, but the modeling practices can be rationalized nonetheless on cognitive grounds, as meaningful steps in the direction of predictive models. Such insights help demonstrate the useful role that cognitive approaches can play for philosophy of science in our attempt to unpack, and discover the rational basis, for scientific practice.

## Cognitive dimensions of model-building in integrative systems biology

We begin this section with a brief description of the field and the nature of the problem-solving tasks that confront our modelers, before detailing some of the specific kinds of inferences modelers need to make during the model-building process and the cognitive processes they use to make those inferences.

Two overarching aims of modern systems biology are (1) to build detailed large scale representations of biological systems (Kitano 2002) and (2) to discover any design or organization principles that characterize the components of systems (Alon 2006, 2007). In our labs the first goal is the predominant goal of individual researchers. Our labs specialize in building ordinary differential equation models (ODE models) of gene regulatory, cellular metabolic and cell signaling networks. Variables in the model describe concentrations of each metabolite in the network in an individual cell (“pathway”). These models are run to simulate the changes to the concentrations of metabolites in a cellular network over time, where each metabolite pool interacts with certain other metabolites, represented as its neighbors in a network. In general our modelers aim to produce models that make good predictions of the dynamic relationships between certain variables in the model, and are thus robust in performance with respect to parameter and initial condition variations. We label such models “predictively valid”.

One of the central assertions of modern systems biology is that in vitro approaches of traditional experimental molecular biology are insufficient for understanding and controlling the causal properties and behavior of biochemical networks. While experiment may reveal local causal interactions between molecular elements, biochemical functions tend to be controlled and orchestrated through large scale networks (networks with wide boundaries) of large size (involving many interacting elements). These networks tend to function through nonlinear interactions, such as feedback loops. As a result the causal properties an element of a network has are dependent on interactions happening upstream and downstream in the network. These features explain in part why particular systemic diseases like cancer or cystic fibrosis have proven so difficult to treat (Hood et al. 2004). Complex networks such as these generate robustness and redundancy, and nonlinear sensitivity to certain parameter changes, which make them difficult to control, and also give rise to variability across individual cells and organisms. As such only simulated quantitative models of these networks can capture networks at the scale and size required to identify variabilities and predict network behavior in response to perturbations accurately enough to know how to intervene on them effectively. These high fidelity models are needed, for instance, to help us estimate the right drug combinations and dosage for any individual to control a disease effectively. It is important to point out, however, that such simulative modeling is only possible because of the massive amounts of experimental data made available on-line in curated databases, the development of new experimental methods for large-scale data collection, and, of course, experimental validation of model predictions.

The field of systems biology is nonetheless heterogeneous in its approach to using computation, ranging from highly computational approaches using big data technology (high-throughput systems) and data mining algorithms in order to reverse-engineer system structure (often called top-down) to the more bottom-up techniques that work with data accumulated by experimental molecular biologists to develop mathematical models that can drive computational simulations of networks (Westerhoff and Kell 2007). The systems biology labs we have focused on are of the latter kind.

### Complex problem solving tasks

Levins criticizes systems ecology as a brute force approach to modeling running counter to his view of what the aim of modeling should be, namely to generate understandable simplified abstract or idealized representations of phenomena (Levins 1966). Systems biologists do rely on key abstractions, particularly the mathematical representations they use of biochemical interactions. One goal is to generate models that are easy to explore mathematically (see Voit 2000). However, given the sensitivity and complexity of biological systems, modelers in the field (particularly those who favor a bottom-up approach) are committed to the view that details matter and abstraction should be minimized or carefully controlled.^5^ Detailed “mechanistic” representations of networks and accurate parameters are required for producing predictively valid models. In this way modelers walk a tightrope between exhaustive and more tractable idealized representations. In the best case scenario, a modeler would start with a well-described or developed pathway of the particular system he or she is interested in (the *pathway diagram* or *pathway representation*), which documents the sequences of all important interactions within a network.^6^ They would decide how to represent mathematically (the *mathematical model or representation*) the various interactions among metabolites and use the data available from experimentalists or high-throughput technology to derive the parameters for those interactions. With suitably rich data the number of undetermined parameters should be low, allowing a straight-forward algorithmic calculation of a best fit.

Unfortunately most modeling situations are quite far from this ideal and the central challenge for modelers is how to put together reasonably robust models under much messier conditions. In the first place, there is almost always a serious deficiency in the data available for model-building. Pathway structures and parameter sets are often incomplete. Modelers have to derive what data they can from the literature in order to both fill in the pathway and estimate parameters. Second, since the data often have to come from different sources derived under different experimental conditions, errors are almost always introduced which have to be corrected for. Even then most modelers are left with large numbers of undetermined parameters. Third, as we have documented elsewhere, collaborative relationships are fraught with difficulties (MacLeod and Nersessian 2013c, 2014). For instance, modelers who collaborate are rarely able to get the experimentation they need performed at critical points in the model-building process. Fourth, ISB lacks domain theory that can be relied upon to instruct modelers on how to go from a given data set to a good representation. The quality, quantity, and type of data can all vary substantially, resulting in a variety of situations that are too broad and too various to be fit to one canonical approach. Most often researchers have to choose how to represent interactions and what aspects of pathways to model given the nature of the data (Chandrasekharan and Nersessian 2015; MacLeod and Nersessian 2013a, c). This is often an intensive process of figuring out precisely what they can represent reliably with the data available and adapting the problem they are trying to solve to fit these data constraints.

Finally the complexity of biological networks amplifies the difficulties of finding good representations. Complexities include the facts that networks contain frequent feed-forward and feedback effects and that many elements play multiple roles in a network. Fitting a mathematical form to such highly nonlinear systems is a complex problem. Adding new structure for instance may be necessary, but this requires predicting what effects a modification to the pathway representation will have and where the modification needs to happen to resolve the problem, neither of which may be obvious. Further given the unpredictability of changes in parameter values in the model it might be hard to isolate regions of the parameter spaces to search for finding good fits. This leaves much work to algorithmic processes of parameter fitting, but these run up against computational constraints, and such processes are unlikely to find the best fit in such circumstances. The result is that representing accurately even only specific dynamic relationships in the networks can be a highly time-consuming and highly iterative process.

### Model-based inferences in model-building

To be able to build models, modelers typically have to rely on the dynamical behavior of their models to make inferences about how to proceed in building the model, such as what elements to include in the pathway representation of the biological system. Modelers need to infer for instance,

1. Structural and parametric errors in models;
2. The nature and form of missing network structure (network elements, relations);
3. Dominant dynamical variables in the network.

#### Inferring errors

In the course of modeling it is typical for modelers to discover, or at least come to suspect, that the mathematical and pathway representations they have developed from background models and from information in the literature are not adequate to get an ODE model which fits the system they are studying under plausible parameter ranges. One of the central tasks of the modeler in order to move forward is to infer precisely the points in the mathematical representation where these errors occur and what type of errors they are. Errors can be related for instance to critical missing elements and interactions in the pathway, the adequacy of mathematical representations of interactions between elements, or parameter values. This kind of inference often depends heavily on model-based reasoning: in particular using the model simulation to infer the location of an error.

For instance G16 was given a task by the lab director G4 to improve upon a certain mathematical model (a set of coupled ODEs) of glycolysis in the bacterium *lactococcus lactis*. Once a better working model was in place she hoped to be able to “incorporate pH effects in the model, and ultimately genetically modify L. lactis to synthesize a required drug and withstand stomach acid in order to deliver the drug to the intestine, thereby circumventing the discomfort and side effects of currently available intravenous therapies” (poster presentation). However even trying to produce a better model over the one available proved a highly complex task. One of the central problems with the original model was that it failed to predict correctly, when fit, the existence of a known peak in the catalyst FBP for different environmental glucose concentrations. The model for instance gave a peak at initial values but not at 20 mmol of glucose, or 40 or 80.

The long term goal is making this bacterium survive the acidity of stomach and somehow preserve the pathway for the lower PHs. But right now we are trying to model it to improve the model as much as we can. So right now the question I am answering is like how to change the model in a way that it captures some specific effect. That effect being when you input more glucose into the system the peak doesn’t go off it… the peak is always same thing. (2011-08-09-i-G-G16/168)

She had a certain amount of good but patchy data to work with from a collaborator G7 (a postdoc in lab G who was an experimentalist transitioning to becoming a modeler). These data only gave information on some parameters, meaning that many would have to be fit or estimated in other ways. By observing the effects of different parameter changes through the model using certain representations (see for example Fig. 3) and via simulation she was able to draw the conclusion that under no reasonable modifications of the existing model could it be made to reproduce the right peak behavior. Further she was able to diagnose precisely where the likely errors were to be found in the model.

I find glitches in the model, and why is it that, for example, sometimes I was trying to model something and then it wasn’t getting better. And when you look at more closely and there’s no way it can get better because it depends on two things, and those two other things, for example, are increasing. So you can never get it decreasing for a period of time from those two. Maybe something else has a role that I haven’t taken into account. (2012-08-31-i-G-G16/65)

In this case G16 managed to isolate two upstream elements in the model which were both interacting with FBP so as to prevent FBP decreasing under greater glucose concentrations. Researchers often refer to this kind of analysis and understanding of a model as having a “feel for the model” (Voit et al. 2012a, b). It consists in developing an ability to understand what role model components are playing and how they serve to constrain the dynamical behavior the model can produce (see Sect. 3). Our modelers rarely have access to data or new, targeted experimentation which can help pinpoint precisely where their assumptions might be wrong (MacLeod and Nersessian 2013b). Instead the mathematical model is their only platform. Modelers try various simulations based on the experimental data they have (which usually maps the relations between just particular variables) over different potential parameter sets to try to infer the mathematical limitations in the model which might be causing an error.

#### Inferring new network structure

Once errors have been detected and localized, modelers need to hypothesize what might be missing. To do so requires an understanding of how their model functions but also, more particularly, the effects of changes to the model. This in turn requires a skilled knowledge of mathematical relationships. Being new to modeling in biology G16 was unsure how to modify her model to produce the right behavior, given her lack of biological knowledge. There could have been a missing element in her model, but she struggled to identify what is was herself. G7 gave her some important advice. “He suggested I try to work like—think about it mathematically. And when I make it right mathematically try to see why this happens. What is the explanation behind it.” (2011-02-07-i-G-G16/104). Finding an approximate or appropriate mathematical relationship would help use the model to narrow the biological possibilities.

G16 decided to experiment with the interactions governing the upstream molecules and their network neighbors to see if she could dampen their influence in the right way. She thus toyed first with a more complex Hill-function to represent catalytic interactions then switched to a step-function because of its tractability.^7^ For a specific set of such interactions she hypothesized that a Step-function interaction (something that approximates a Hill type of catalytic interaction) rather than a logistic function (for instance Michaelis–Menten or power-law) would have the desired effect of correcting the dynamics in a straightforward way with minimal parameter requirements. Checking the potential validity of the hypothesis was nonetheless a complex process. It was not just a matter of running the model, but of refitting the parameters to see how well a good fit solution worked and inferring back to the validity of the hypothesis. In this case it produced a reasonable result which G16 thought was a good candidate for representing a specific interaction in the right way given their performance, pointing the way to underlying unaccounted for biological influence on that interaction. Of course not all inferences made this way will be correct, as G16 noted, “…these are vague ideas. Maybe none of these work. Right now I should run it for the assumptions I have, just collect the curves.” (2011-02-07-i-G-G16/132). Given the complexity it is surprising that accurate inferences are able to be drawn through such processes. Nonetheless in our labs researchers have a solid history of making good inferences that lead to robust results and experimental validation. Some of these inferences can be quite novel.

**Fig. 1:**
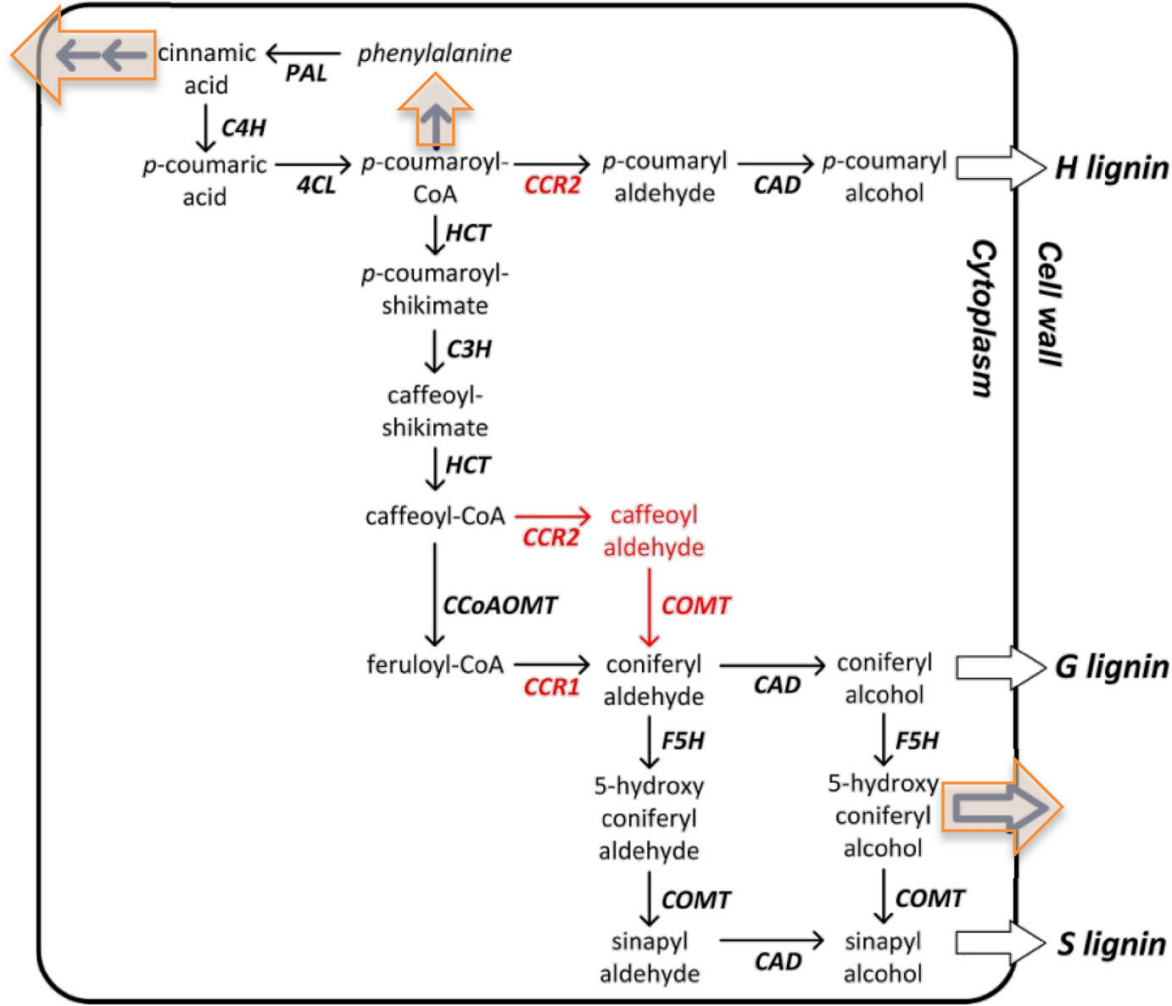
G10’s modified pathway diagram for the alfalfa lignin system. These molecular pathway diagrams display sequences of chemical transformations within a cell (or across cell-boundaries) which give rise to a particular biological function (in this case lignin production, represented as H, G and S). The biochemical elements and their interactions can be translated directly into a mathematical model by which nodes represent the concentrations of the chemical representations and arrows the rates of reaction. G10 assembled an original “wild-type” diagram based on known results provided to him by his collaborators. Various elements were added to the original by him, such as the arrows connecting p-coumaryl CoA to phenylalanine, and cinnamic acid to the environment, the latter signifying a loss from the system. Models built based on the original diagram were mathematically incapable of producing accurate behavior when inputs were perturbed out of equilibrium suggesting to him that some regulatory mechanisms controlling extra flux had not been factored into the current biological picture. He identified additional interactions which would resolve the extra-flux, in particular those arrows connecting p-coumaryl CoA to phenylalanine, and cinnamic acid to the environment

Consider the case of G10, who was modeling lignin synthesis in two plant species (Chandrasekharan and Nersessian 2015 details this case). Lignin, a structural material in cells, interferes with attempts to get plant metabolism to produce biofuel chemicals and G10’s goal was to try to understand how to control lignin production to make biofuel production more efficient. His experimental collaborators provided him with limited data and he had to assemble the synthesis pathways for both species (poplar and alfalfa) himself. His original pathways represented nearly a 20-year consensus of collected biological opinion on lignin synthesis. However, particularly in the case of alfalfa, it became clear to G10 that the set of interactions and elements as described in that pathway representation plus the available data could not produce the right mathematical relationships between the particular variables he was interested in. The original model was built for the wild-type system at steady-state, and such a model was not necessarily capable of handling successful manipulations of the system. Indeed G10 discovered that the original pathways were mathematically incapable of producing accurate behavior when inputs were perturbed out of equilibrium suggesting to him that some regulatory mechanisms controlling extra flux had not been factored into the current biological picture. Using the pathway diagram to help identify plausible network additions, and then perturbing the mathematical model through the aid of simulation to check these additions, G10 was able to hypothesize sets of additional fluxes to the model which would eliminate the excess flux in the appropriate amounts and then choose among them according to biological plausibility (see the highlighted arrows in Fig. 1 below). For instance a surplus in the model of p-coumaryl CoA could be handled mathematically in a biologically plausible way if some of that flux was removed to the production of phenylalanine and eliminated from the system through cinnamic acid leaving the cell. These he translated to more precise mathematical modifications that would relieve the system. In his words “this is an important piece of knowledge that comes from the model,” through understanding its dynamics.

**Fig. 2:**
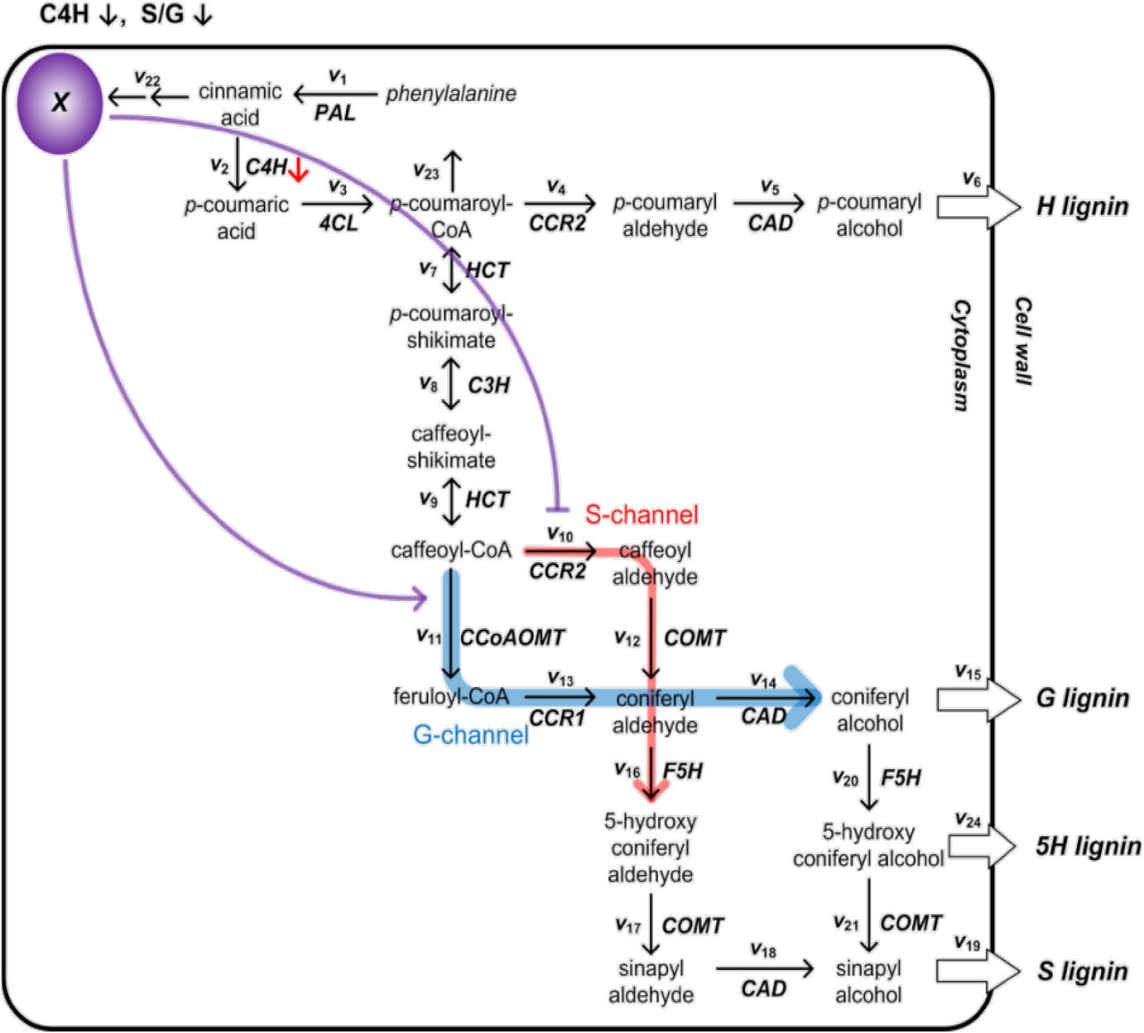
G10’s drawing of the pathway diagram for the lignin system with unknown molecule X added with feedback and feed-forward relations regulating the S and G channels respectively. The S-channel serves to down-regulate S-lignin production and the G-channel up-regulate G lignin. G10’s model thus acquired a mechanism for producing the lower S/G ratio predicted in the data. X also accounted for the excess cinnamic acid G10 had originally hypothesized as leaving the system

Further, using information he had on down-regulation and up-regulation of particular variables and their effects on G and S lignin production, G10 reasoned that G and S lignin production were happening in ways outside of what was mathematically possible within the model, despite his flux additions, namely the model was giving too high a value for the ratio of S to G. However from an understanding of the dynamics in his model G10 inferred that the easiest and most efficient mathematical way to resolve this problem was to hypothesize another element in the network (see Fig. 2) being produced from the excess cinnamic acid. This element would be selectively regulating the pathways or “channels” responsible for generating S and G lignin. He called this element ‘X’ because he lacked sufficient biological knowledge to hazard a guess as to what it might be. If cinnamic acid actually produced a substance X which both up-regulated G-channel flows and down regulated s-channel ones, then a model could be generated which produced very accurate dynamical, not just steady-state, behavior.

So this is actually the biggest finding from our model. So by adding this reaction you can see that we hypothesize that there is another compound that can give a regulation….give a feed forward regulation to other parts of the pathway. (2011-04-12-i-G10/20)

His postulation of a here-to-fore unknown metabolite in the lignin pathway is a novel inference derived from a very good understanding by G10 of quantitative movements within the network model and how to control the numbers effectively. The inclusion of X led to a robust model on his part that supported his hypothesis, and is the sort of outcome considered by systems biologists to be an excellent example of the investigative power of model-building. In this case his hypothesis was borne out by his experimental collaborators and hailed as a major discovery.

#### Inferring dominant dynamical variables

Another kind of model-based inference our modelers are required to make is of the dominant dynamical relationships operating in the systems they are investigating or at least with respect to functions of the system they wish to account for. Such inferences allow modelers to simplify their models by removing or treating as constant particular variables (i.e. metabolite concentrations), or fixing their governing parameters arbitrarily with easy to handle values. Ultimately the parameter-fitting problem is reduced as a result. Finding parameter fits can be impossible otherwise. Parameter landscapes for nonlinear systems with too many unfit parameters will generate many local minima. Searching through entire parameter spaces using available methods is too computationally intensive and too likely to find inadequate minima. Getting a successful parameter optimization is thus a driving factor in the decisions modelers make about how to represent their system.

The main techniques of extracting dominant relationships come under the heading *of sensitivity analysis*. The term and the many specific methods are inherited from engineering. Sensitivity analysis has a number of functions apart from just identifying dominant relationships, such as discovering errors in models. In terms of identifying these relationships, sensitivity analysis involves processes and techniques by which modelers scan the models to find out which parameters have the most effect on the model and which have the least. It is thus again a type of model-based inference. The ones which have minimal effect can in theory just be removed from the network representation or given constant values—often just 0’s or 1’s—to simplify even further the mathematics. As in the case of inferring new structure, inferring dominant relationships in a network requires insight into how the available mathematical representations work (assuming they are for the most part accurate). Variables are less dominant the less effect that changes in their governing parameters have on network dynamics.

Sensitivity analysis is often performed locally by studying the mathematical structure of the model(s) and studying effects of changes in individual parameters and following the effects of those changes through a network using pen and paper. Researchers often build mathematical arguments based on model structure (examining for instance partial derivatives) to justify removing a variable from a network or using trivial values for its parameters or “off-lining” it by treating its output as constant. For instance according to a lab C modeler, C7,

and then, that’s where the, you know, the trick comes in, the good modeler would know how much to restrict the system so that he has most of the things that are known—and very few things that are unknown—and those can be tested. You find points in the system that are more sensitive to changes… So, if you change the less-sensitive points, it doesn’t affect the output as much. So, what you can do is find what’s more sensitive, if that is unknown, try to tweak things there. (2009-04-i-C7/76)

If the system is too complex for such step-by-step exploration of sensitivities then modelers will often turn to a more global computational method. G10 for instance employed a statistical strategy, a type of variance-based analysis, running his models with a thousand different parameter sets (sampled using Monte Carlo techniques) and calculating the Pearson correlation coefficient of each parameter with the S/G variable he was particularly interested in. He used this process to make a statistical argument about which parameters were most significant with respect to that variable, trivially setting the rest. Such methods avoid having to make inferences about sensitivity directly, but they are computationally intensive and statistical techniques will average out sharp differences in the effects of particular parameters in different parameter domains. The only way to discover and become aware of these is with more local investigation of the model.

## A “Feeling for the Model”: simulative mental modeling and distributed cognition

The exemplars considered provide some insight into the cognitive dimensions of ISB modeling: how methodological and representational choices in model building are aimed at reducing a complex problem to one that is cognitively tractable and how computational model-building and simulation processes support inferences that lead to novel hypotheses about phenomena under investigation (e.g., biological pathway elements). In this section we propose a cognitive account of how they are able to draw these inferences.

All these inferences depend on being able to understand to a degree how the model operates and estimate what the effect of changes might be. Modelers necessarily rely on developing the ability to filter good hypotheses out of the large collection of hypotheses so as to select which time-consuming but risky modifications to try. Modelers themselves often talk in more general terms about the “understanding” or “insight” necessary for modeling. Our data support the modelers’ claims that such understanding is critical to their success and to their ability to get more insightful results.

Speaking of the need for insight as opposed to just raw optimization methods used by some systems biologists, G16 states,

I don’t like optimizing because—by optimization you would think, well just use one of the optimization methods—genetic algorithm this and that. It’s not like that because it’s a very huge system, usually. Like a lot of variables if you just use this and that optimization method, it’s not going to work….the error surface is like—has a lot of like—local minima…you will just get stuck in one of them. So you gotta have insight, then there’s a lot of—as [lab director] puts it, ‘elbow greasing’. (2012-02-15-I-G-G16/86)

Optimization is, as she calls it, a “blind process”.

Cause it’s easy to fit everything in and say, this is how it works. But then if you really want to get the results afterwards, like have the model—let the model have the predictive power you want it to have, you gotta be sure about what you are doing. (2012-02-15-I-G-G16/260)

In general, processes of developing this kind of understanding—of being “sure”—are slow.

So when you get here, you’re like very frustrated. Like, nothing is known to any extent [with emphasis]. And then you think like—I don’t think you can get any truth out of the system. That’s what you think in the beginning…after a while, you know what to expect and you know that kind of thing is not gonna—you can reason that that kind of thing and that error in there is not gonna effect the whole system like that. (2012-05-30-i-G-G16/96)

At the heart of these notions of insight and understanding is the notion used by modelers we identified above of having a “feel for the model.” We analyze it as a kind of understanding of the dynamical behavior of a model which enables the modeler to locate errors and also infer what changes will occur when adding specific new elements or removing variables. Given the nonlinearity and complexity of these models it is no easy task to acquire this understanding. However, the notions of having a “feel for the model” or “insight” admittedly sound vague and somewhat esoteric—the kind of notions philosophers shy away from. However it can be given a more concrete sense. In cognitive terms our data suggest that the ability for modelers to generate inferences from these complex networks requires two cognitive components: simulative mental models and cognitive distribution (Nersessian 2002, 2008, 2009).

### Simulative mental modeling

Although there are many notions of ‘mental model’ in the literature, Nersessian (2008) has constructed a “mental modeling framework” that provides a cognitive basis for such model-based inference science. Notably, there has been an important line of theoretical and experimental research going back to the reissue of the 1943 book on explanation by Kenneth Craik (1967) which focuses on specific processes of dynamical and mechanistic mental modeling. This research provides a cognitive basis for understanding what modelers do in the cases of inference we have mentioned above. Nersessian provides “minimalist” description of a simulative mental model in the form of a hypothesis about reasoning as follows:

…in certain problem-solving tasks, people reason by constructing an internal iconic model of the situations, events, and processes that in dynamic cases can be manipulated through simulation. Such a mental model is an organized unit of knowledge that embodies representations of spatiotemporal relations, representations of situations, entities and processes, as well as representations of other pertinent information, such as causal structure (2008, 128).

The descriptions and self-reports we collected from a range of modelers in both lab G and lab C suggest that our modelers build mental models of their networks which allow them to mentally simulate limited aspects of network dynamics in order to identify errors, explore new hypotheses about structure or parameters, and identify dominant variables. When they communicate with us about what is happening in their system models and how they perform these various inferences, they do so using such mental models. Experimental research in cognitive psychology and AI research on dynamic mental modeling in lay “scientific” thinking is extensive and we extract only the most relevant findings here. This research has identified specific features associated with mental simulations of physical and mechanistic models such as multiple pulley systems. These simulations are,

1. Qualitative (Roschelle and Greeno 1987; de Kleer and Brown 1981).
2. Piecemeal (Roschelle and Greeno 1987; Hegarty 1992, 2004; Schwartz and Black 1996).
3. Supported by background knowledge in long-term memory (Roschelle and Greeno 1987).
4. Coupled externally with visual and other representations (Hegarty 1992; Hegarty and Steinhoff 1997).

As we can infer from their descriptions, the mental models our researchers construct appear to manifest these properties. The mental models involved in the specific inferences we have discussed above are typically not quantitative. These models track and record only the qualitative behavior of variables. Our modelers orally express their thought processes in the language of “increasing,” “decreasing,” “inhibiting,” and so forth. Consider again this statement from G16,

I find glitches in the model… And when you look at it there’s no way it can get better because it depends on two things, and those two other things, for example, are increasing. So you can never get it decreasing for a period of time from those two. Maybe something else has a role that I haven’t taking into account.

Underlying G16’s inference is a mental representation of interactions in the network, which she uses to make the counterfactual claim that under no circumstance could any manipulation of these variable produce the right behavior in her target variable. She derives the claim by manipulating various variables (mentally changing their values). Reasoning with these structures provides modelers the ability to test mentally what the effect of modifications to the mathematical model might be by mentally simulating them.

To do this however modelers work with mental models of only limited sets of variables at any one time. G16 for instance describes only tracking a few elements in the description she gives of her reasoning. This is consistent with the results of Hegarty’s (1992) experiments on subjects solving pulley system problems. Hegarty found that the attention of participants was confined mostly to directly connected pulleys at any one time. Systems were worked through step by step. The self-reports our modelers give of how they operate also suggests they do not reason with many elements at any one point in time, but focus on directly interacting elements in a network. However these elements do not need to be contiguous. Information derived from the pathway and the mathematical equations governing the network can be used to build up information on interactions among more remotely located elements, which can then be used to simulate the effects of interactions between these elements. The modeler builds expertise and knowledge of how to group the effects of interactions (see Hegarty 1992).

The process of building up a feel for the model is iterative and intensive. It requires, among other things, finding ways to familiarize oneself with how the model works in order to interpret the qualitative effects of the various quantitative mathematical relationships (Roschelle and Greeno 1987). This kind of process is sometimes called *envisioning* (de Kleer and Brown 1981). G16 describes her process of building an intuition of how an equation works:

So the thing is—when you want to solve a mathematical problems, you gotta—sometimes you use numbers and try numbers, something to give you a feel of—like intuitively how this, for example, equation works and all. So I’m trying out numbers and then trying to make the steps kind of discrete—like sort of a state machine, kind of thinking like we’re in this state. And then now this much is going to this other metabolite pool and then at the same time we have less of that. So I’m trying to see what the constraints are by actually like doing a step-by-step sort of thing. (2012-08-31-i-G-G16/63)

In general, envisioning or building mental representations of model relationships requires a background understanding of how to interpret mathematical equations, but also, in the case of complex equations, requires processes that can visually unpack model dynamics and allow them to be more readily interpreted. G16 showed us an example of her process of visually tracking values in her model with pen and paper representations as she was working on the problem. Model variables were being solved numerically by her at different time steps, so she could track behaviors in the model directly. This process was likely critical to her ability to build mental models of relationships in the network she was studying, and in turn to draw various inferences about the location of errors, potential modifications and dominant relations.

### Coupled cognition and the role of computational simulation

**Fig. 3:**
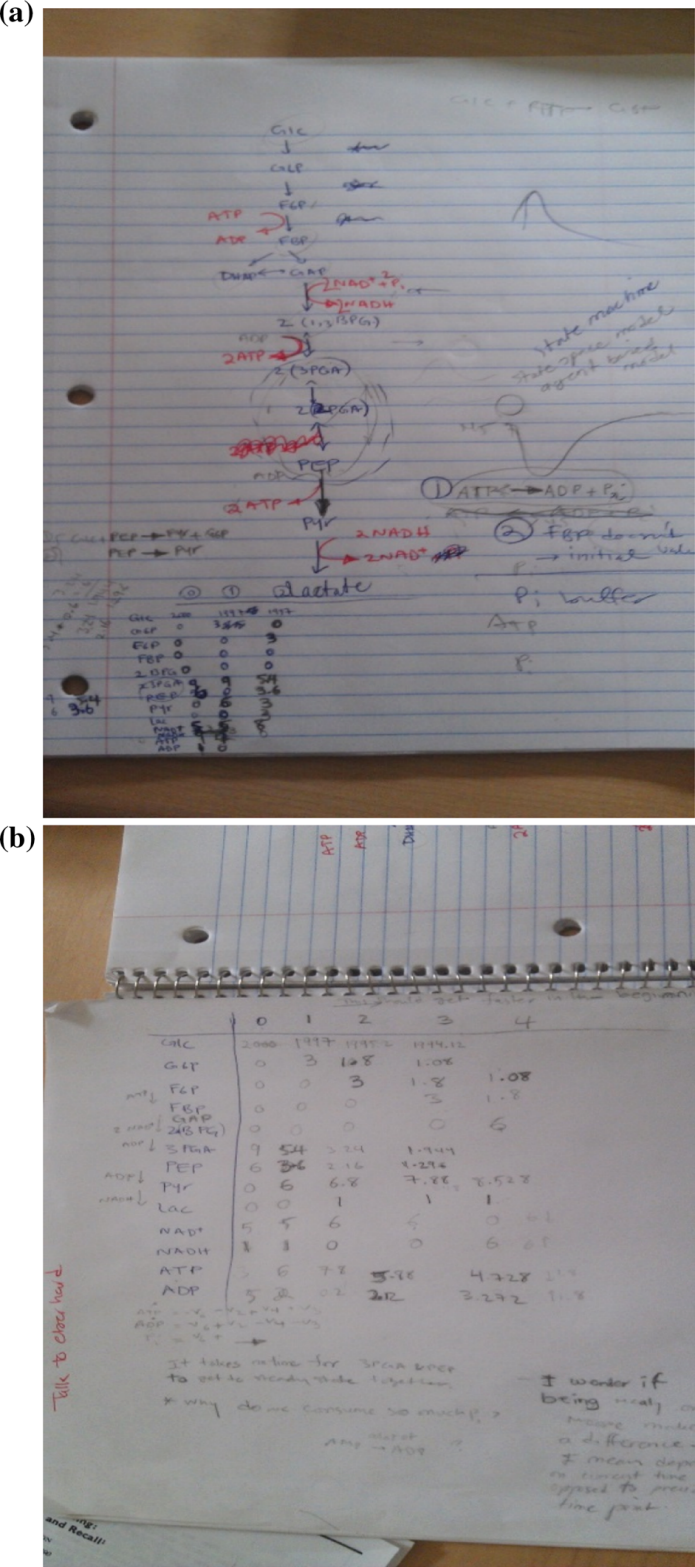
G16 working towards “getting a feel” for the model or envisioning relationships using pen and paper representations of numerical information to interpret the effects of the equations. **a** The pathway diagram she worked with in this instance. Around the diagram are various notes to help her interpret network dynamics using the diagram, including a numerical table tracking the evolving concentrations of elements at sequential time-steps. **b** A larger version of such a table including more elements of the pathway. G16 manually calculated the concentrations of biochemicals in the network in order to visually represent how fluxes move through the network, which provides her information on qualitative relationships in the network. Such diagrams could also be used to observe the effects of parameter modifications

The account outlined above suggests an important role for simulative mental modeling in model-building processes in ISB at the heart of many of the inferences model builders need to make to improve their models. It also suggests an important role for external representations, as we saw through G16’s use of certain visual representations to build her own mental models. At the same time modelers like G16 rely on pathway diagrams (see Fig. 3a, b) during their reasoning processes to provide a visual reference or scaffold for manipulating model internally without having to represent or keep track of all structure purely mentally.^8^ As such Nersessian (2002, 2008, 2009) has argued, in accord with the cognitive science research, that mental modeling of dynamical processes is often *coupled* with pen and paper drawings (diagrams, sketches, graphs) and with physical simulation models by means of which scientists reason about in vivo phenomena (Nersessian and Patton 2009; Nersessian 2009). Chandrasekharan and Nersessian (2015) have argued the case for coupling with computational representations. Together, drawing inferences by means of these coupled systems of mental and artefactual models comprise what Nersessian (2002, 2008) calls “simulative model-based reasoning.” The notion of coupling extends the framework of distributed cognition (Hutchins 1995) which investigates the ways in which external representations used in problem-solving processes perform cognitive functions, thus creating a distributed cognitive system comprising humans and artifacts. The focus of distributed cognition has been on the use of existing artifacts, and our research group has been extending the focus to the building of representational artifacts, such as physical and computational simulations models (see, e.g., Chandrasekharan and Nersessian 2015; MacLeod and Nersessian 2013a; Nersessian 2009, 2012; Osbeck and Nersessian 2006).

In most cases of modeling there is a limit to what can be done with visual pen and paper representations. Computational representations are the central resource for handling complex systems that exceed mental modeling capabilities. Computational modeling is closely coupled with simulative mental modeling in model-building practice. Together they form a coupled cognitive system relying on both components to perform essential calculations and generate inferences neither alone could. For instance, the computational model provides the modeler:

1. Visual representations of complex model dynamics that can be translated into qualitative relationships;
2. Piecemeal and selective visual representations that can be represented mentally within cognitive capacities;
3. The ability to check or calibrate the results of mental simulation and correct their mental models; and
4. The ability to test their mental inferences about network structure and behavior.

The value of computational modeling and simulation for this coupled cognitive systems stems from the ability of computers to perform complex calculations that are cognitively intractable for the human agent and from the ease with which computational representations of complex system can be visualized in the way modelers require (for instance, providing visual graphs to track just specific relations). Further the simulative capacity of the computational model provides the ability to implement manipulations quickly and efficiently so that modelers can run through plausible options or hypotheses in quick succession. In these ways information is exchanged back and forth between both components of a hybrid computer–human cognitive system, and the overall cognitive benefit is to extend human cognitive capacities so that accurate inferences about how to improve complex models of complex systems become possible. Diverse network behaviors in response to parameter variations or at different time points, as a result of nonlinearity, can be identified through computational simulation and partitioned into families of mental models representing network relations under different conditions or at different times. This is consistent with the piecemeal notion of mental simulation discussed above. So for instance situations in which feedback produces oscillations can be bracketed from those in which it produces more linear behavior and treated somewhat separately. If two variables of interest are placed far apart in the network simulation their relations to one another can still be detected leaving out any complex intermediate interactions. The gradual development of the coupling enables the modeler to develop detailed understanding of the pathway’s dynamical behavior contained within families of mental models through running thousands of computational simulations, using many parameter combinations, and analyzing system dynamics for each simulation.

## Cognitive constraints and methodological choice

Simulative mental modeling and distributed cognition, together, can provide a cognitive account of how model builders generate the inferences they need to construct models of complex biochemical systems. They account for how modelers are often able to produce quite substantial discoveries, with only the barest biological knowledge, during the process. In general modelers are able through these techniques of producing models that capture at least some of the dynamics of a system well. But the models produced are usually far from complete, and often fall short of the goals individual models have, not to mention the field at large. As mentioned earlier, part of fundamental rhetoric of systems biology is that control is distributed over large scales, so large-scale models are required in order to capture the control structure of actual systems. Molecular biology, with its focus on local interactions, can never develop that kind of insight. Mathematics and computation are essential to deriving these relationships from complex systems. However systems biologists using the kind of bottom-up strategies our labs use do not work with systems of the desired size (number of elements of a network included) and scale (the inclusiveness by which boundaries of the network are drawn), but with smaller sub-systems or simplified systems. In fact the kind of models produced do not enable researchers to meet another other goal of systems biology either, which is to extract the design and organizing principles underlying biological networks. As Voit et al. put it, “If one would survey all computational systems models in biology, published during the past decade, one would find that the vast majority are neither small enough to permit elegant mathematical analyses of organizing principles nor large enough to approach the reality of cell or disease processes with high fidelity” (2012a, b, 23).

Systems biologist have proved mostly incapable at present of handling systems of large enough scale to get models that are predictively valid and reliable enough in particular for medical decision making (see Voit et al. 2012a, b). On epistemological grounds the models built fall short of what they are built for, and indeed a philosophical analysis that only analysed the epistemological structure of these models would find them unjustified. We believe that the kind of cognitive account we are giving of model-building processes also helps explain partially at least why modellers choose to construct models of insufficient size and scale given the goals they often set out with (Sect. 4.1). Further a cognitive approach can also help explain and rationalize the size and scale at which representations are being constructed within the field as a case of “mesoscopic modeling” (Sect. 4.2).

### Constraints on simulative mental modeling and their implications for modeling

Although computational modeling can extend the ability of modelers to deal with more complex systems, the human agent is notably a component in the model-building processes. The human component has not received much attention in discussions of distributed cognition to date. But it is the human component which often provides the rate-limiting step on the level of complexity that can be addressed through these practices. It is well known that people in general are not good at building and using causal mental models—even less so with nonlinear systems (Doyle et al. 2007). In the case of simulative mental modeling one can identify a clear constraint in the form of working memory (Hegarty 1992). Humans can only keep a limited set of interactions in their minds at any one time. Visual representations and computations help extend this, but ultimately it is the human agent that has to draw an inference by processing the information before him or her. If this information involves too large a set of factors or these elements are interacting in too complex a way to be qualitatively processed into descriptive units of information such as “increasing”, then the task will be too difficult.

This factor puts implicit limits on the complexity of the networks with which modelers can deal. Particular complex features of networks introduce many factors that need to be processed simultaneously in order to make inferences. These often involve quantitative sensitivity that is much harder to decompose into small packets of qualitative information even with the aid of computation. These factors include for instance, feedback relations, competitive reactions and multiple network functions of elements, many interactions at any node, and long chains between relevant variables which will include more of these types of interactions to process.

The more of these interactions, the harder mentally simulating a set of network components in order to improve a computational model is likely to be. Some of these kinds of issues can be dealt with by simplification processes, but as stressed many of those kinds of simplification processes (e.g., inferring sensitivity) depend themselves on judgments and arguments constructed through mental modeling. Broadly speaking it is plausible to expect then that human cognitive constraints set limits on the complexity of models that can be reliably constructed and thus on the complexity of the systems that can be represented. Of course there is no way to articulate precisely how to pick systems that meet these constraints. One way to control complexity is to manage network size and scale and keep network representations relatively small through careful selection of what networks to model and which of those to include in a model at the outset. Smaller, more limited pathways reduce the demands on working memory for debugging and model-based inference.

Modelers themselves correlate scale with complexity and see it as setting limits on their ability to get the necessary insight into how their models work. For instance G70, an experimental collaborator with the lab G director (G4), reported this reaction from G4 after handing him a large network to model.

But I think he’s (G4) been in the real world long enough doing this systems stuff long enough that he knows to start small… so when I first came to him, I had the proteomics systems. We’ve seen about 10% changes in about all the changes in all the systems of the CF cell versus a non-CF cell. Now when you think about the number of systems that are in cells, 10% changes in all of those systems… is a considerable amount, I mean that is a lot of information. So when I first went to… he’s like you are diluting yourself. So then we decided to start with glycolysis and the pentosphosphate pathway of the Krebs cycle… to narrow it down to energetic pathways that are very well modeled.

Instead of trying to build a model of such a large, intractable network, G4 directed G70 towards the most cognitively reasonable strategy: using small models that were already established and building outwards with those. In general modelers choose not to work with entire sets of functional interactions that govern a phenomenon but only with, hopefully, significant subsets. They choose not to incorporate all interacting elements. In many cases they just do not know what all the relevant interactions are, as we have seen. Further they choose to model interactions in less accurate but more mathematically tractable ways. With an approximately accurate result they have leverage for improving their model in a more piecemeal but tractable fashion by increasing network complexity. This kind of strategy has been called a “middle-out strategy” (Noble 2008). It forms an essential component of the cognitive strategy of “mesoscopic modeling.”

### Mesoscopic modeling: a cognitive strategy

Cognitive constraints are not the only constraints modelers face. They also have to deal with computational and data constraints, which all play a role in the decisions modelers make. But if our description of the cognitive processes underlying model building is on target, it is reasonable to think that cognitive constraints set plausible limits on the network size and scale modelers can handle. That said, an awareness of these cognitive constraints and cognitive capacities also provide a basis upon which to rationalize preferences for building smaller scale more abstract representations. Some systems biologists argue in explicitly cognitive (rather than epistemological) terms that the strategy of building smaller representations enables modelers to work within cognitive constraints towards the eventual construction of the larger models they need.^9^ They label this practice “mesoscopic modeling” (Voit et al. 2012a, b).

Voit et al. cite in particular developing and maintaining an understanding of the system as the critical motivation for restricting models to mid-size models. These models provide a, “coarse structure that allows us to investigate high-level functioning of the system at one hand—and to test to what degree we understand, at least in broad strokes, how key components of a biological system interact to generate responses” (Voit et al. 2012a, b, 23). This basic understanding provides insight into how to recognize deficiencies in the model and expand upon them to give more complex and accurate representations. The basic motivation then for treating mesoscopic modeling as a sensible and reasonable practice for modelers to pursue at the outset is cognitive tractability, so as to keep the modeling process within the ability of modelers to comprehend the model and recognize efficient strategies for its improvement. With this comprehension the model can be scaled up in a middle-out fashion.

Voit et al. argue that the process of building out models rely on basic human learning processes.

This strategy of locally increasing granularity has its (ultimately unknown) roots in semantic networks of learning and the way humans acquire complex knowledge. As a trivial example, consider how we learn about fancy cars. Although infants typically start their learning process with examples, rather than abstract categories, they soon begin to distinguish static items form things that move, eventually learn to differentiate between living and engineered things that move, and become able to distinguish cars from trucks. Later, we begin to distinguish between cheap and expensive cars, and in some cases we learn to tell the year a car was made even if the differences between models from one year to the next are subtle. This hierarchical learning is very effective, because we are able to start simple and add information as we are capable of grasping it. (p 23)

Although Voit and his collaborators focus principally on the value of mesoscopic models for scaling up, rather than for their capacity to be built in the first place, implicit in the kind of understanding or “grasping” they have in mind is the importance of building a “feel for the model.” Models need to be of an interpretable scale and complexity such that the modeler is capable of making good decisions about how to modify them. As we have suggested above the capacities and constraints of simulative mental modeling are the root of this understanding. The strategy of mesoscopic modeling is thus guided by the intuition that cognitive tractability is essential to the model-building process, and thus model scale and complexity matter. Thus, this argument advocating that modelers should build models of limited scale and complexity picks out a cognitive rationale as the primary basis for this choice.

That said, cognition is not the only consideration relevant for this modeling strategy. Mesoscopic modeling has to be sufficiently reliable epistemically. Arguably however the choice of mesoscopic modeling cannot be rationalized easily on epistemological grounds alone. Resulting representations are highly abstract and simplified representations of systems that likely have a loose relationship with underlying system mechanisms (MacLeod and Nersessian 2014). They might represent well particular relationships but much fitting has usually been employed to get those results. The robustness of the resulting models can be difficult to assess. These models do not capture accurately the control structures of biological networks. The likely epistemological requirement at work here is that these models get close enough to what is actually happening in a system such that the smaller scale modifications such as de-black boxing variables into subsystem components will likely improve model performance and accuracy. For this what is required is only a “coarse validation” that demonstrates the model replicates general patterns or trends of functioning in the system. Given the simplifications and abstractions involved it is hard to interpret these models on their own as good or reliable representations. Their potential epistemic value only makes sense given the cognitive value of mesoscopic models and the ability to scale-up such models. An adequate account of methodological choices made in this field needs to go beyond epistemological analysis to at the underlying cognitive motivations.

## Conclusion

In this paper we have tried to outline a feasible cognitive account for explaining problem-solving processes and methodological choices in a computational field like systems biology. We have tried to show some dimensions by which cognition is implicated centrally in processes of model-building, particular inferential processes and, in turn, how these processes are likely limited or constrained. These constraints might well set limits on the degree of complexity models can have in order to be solved using model-based inferential processes. Although many constraints are part of the modeling process, at least some systems biologists see cognitive constraints as the principal reason for limiting model-scales to mid-size mesoscopic models. Methodological choices in the field are thus to an extent at least driven by cognitive limitations and cognitive capacities.

While we do not offer here exhaustive evidence for our account, we do think what we provide motivates the importance of cognitive science for helping to answer traditional philosophical questions about methodological choice and rational principles underlying it, particularly with respect to the current philosophical goal of constructing an epistemology of simulation. Indeed as science with the aid of computation moves to tackle in detail complex phenomena, it can be predicted that cognitive limitations are likely to become increasingly substantial issues for scientific researchers when making methodological decisions. The role of computational simulation in modern science arguably makes human cognitive capacities a much more salient issue, since it allows researchers to go much farther into realms where complexity and cognitive limitations really bite. This is an essential part of the novelty of computational simulation which philosophers need to consider. As Humphreys puts it in the *hybrid scenario* where science is done at least partially by machines, “one cannot completely abstract from human cognitive abilities when dealing with representational and computational issues” (Humphreys 2009, 616; see also Nersessian and MacLeod 2017). Scientists themselves will need to make explicit decisions about what kind epistemic goals are appropriate with respect to model building and, in turn, what kind of cognitive access and control they need to have of the model-building process in order to ensure its success. There is, of course, always the potential for raw powers of computation to take over and automate these processes. But for the foreseeable future, as in the instances explored above, researchers pursuing a bottom-up modeling strategy will need to rely on relatively cognitively rich model-building strategies, albeit with much of the process distributed computationally. As such it is reasonable to expect cognitive factors to play an increasingly prominent role in methodological choice and to anticipate that methodological choice might often have robust rationally warranted cognitive explanations rather than just pure epistemological or ontological ones. Philosophical accounts of simulation methodology need to take cognition into account.

Lastly maintaining a normative focus for philosophy of science in the context of simulation requires much more attention to the practicalities of research (Winsberg 2009, 2010). And here cognitive constraints should bite as well. There seems little point formulating normative proposals if they are not cognitively achievable. Recognizing what is achievable will be aided, significantly, by a deeper understanding of the cognitive processes involved in model-building such as the kind we have considered here.

## Footnotes

1 Italicized quotations are drawn from our interviews of researchers who participated in our study.

2 The ethnographic and interviewing parts of our investigation took place between 2010 and 2014. We then followed up with the graduate student researchers through the awarding of their Ph.D.’s. The last student in lab G finished in 2016. ODE and calculus-based biosystems modeling approaches continue to be a fruitful, especially in the contemporary context of the greater availability of time series data and enhanced computing resources. For a review of recent research using the BST approach of our modelers see (Voit 2013a, b). A range of contemporary practitioners of mesoscopic modeling were contributors to a recent international conference on the state of the field https://www.cecam.org/workshop-1-1269.html.

3 A field of “cognitive science (studies) of science” has been developing since the early 1980 s with philosophers of science, psychologists, and AI researchers participating. Pioneering work in philosophy of science include Darden (1991), Giere (1988), Nersessian (1984, 1992), and Thagard (1988). However since only the more recent work of Nersessian and colleagues that we cite here addresses the practices of computational modeling and simulation, we draw mainly from that.

4 See Nersessian (2008) for a detailed critical analysis of the “mental modeling framework,” and the role of “simulative model-based reasoning” in science and engineering (Nersessian 2009).

5 see however Hetherington et al. (2006) for a conflicting view.

6 See Figs. 1 and 2 in Sect. 2.2.2 later in this paper as examples of such diagrams for a metabolic network.

7 Hill functions models interactions as switch-like, switching rapidly from high to low states as an independent variable like substrate concentration of a reaction catalyst is increased.

8 See also Jones and Wolkenhauer (2012) for a discussion of how pathway diagrams serve as locality aids. These diagrams provide an information rich representation in comparison with sentential representations, which by visual representing relationships, minimizes the amount of search time required to extract relevant information a modeler might need.

9 Some systems biologists and philosophers have proposed ontological justifications of mesoscopic modeling. Bertolaso (2011), Bertolaso et al. (2014), and Giuliani et al. (2014) argue that it is at mesoscopic levels, or scales of network organization, at which functionality emerges in response to higher-level system and environmental constraints. This may well be the case in certain cases. Our researchers do not interpret their results as having this kind of significance in general however. Instead they interpret what they have produced as partially explanatory and of limited application, but nonetheless valid starting points for building more complex representations.

## References

[CR1] Alon U (2006). An introduction to systems biology: Design principles of biological circuits.

[CR2] Alon U (2007). Network motifs: Theory and experimental approaches. Nature Reviews Genetics.

[CR3] Bertolaso M (2011). Hierarchies and causal relationships in interpretative models of the neoplastic process. History and Philosophy of the Life Sciences.

[CR4] Bertolaso M, Giuliani A, Filippi S, Valente AXCN, Sarkar A, Gao Y (2014). The mesoscopic level and its epistemological relevance in systems biology. Recent advances in systems biological research.

[CR5] Chandrasekharan S, Nersessian NJ (2015). Building cognition: The construction of external representations for discovery. Cognitive Science.

[CR6] Chandrasekharan, S., & Nersessian, N. J. Beyond correspondence: How the process of constructing models leads to discoveries and transfer in bioengineering sciences. *Studies in the History and Philosophy of the Biomedical and Biological Sciences***(forthcoming)**.

[CR7] Chandrasekharan S, Nersessian NJ, Subramaninan V, Brown J, Frappier M, Meynell L (2012). Computational modeling: Is this the end of thought experiments in science. Thought experiments in philosophy, science and the arts.

[CR9] Craik KJW (1967). The nature of explanation.

[CR10] Darden L (1991). Theory change in science: Strategies from Mendelian genetics.

[CR11] de Kleer J, Brown JS, Anderson JR (1981). Mental models of physical mechanisms and their acquisition. Cognitive skills and their acquisition.

[CR12] Doyle JK, Radzicki MJ, Trees WS, Qudrat-Ullah H, Davidsen P, Spector JM (2007). Measuring change in mental models of complex dynamic systems. Complex decision making.

[CR14] Giere RN (1988). Explaining science: A cognitive approach.

[CR15] Giere RN, Carruthers P, Stich S, Siegal M (2002). Scientific cognition as distributed cognition. The cognitive basis of science.

[CR16] Giuliani A, Filippi S, Bertolaso M (2014). Why network approach can promote a new way of thinking in biology. Frontiers in Genetics.

[CR17] Hegarty M (1992). Mental animation: Inferring motion from static displays of mechanical systems. Journal of Experimental Psychology. Learning, Memory, and Cognition.

[CR18] Hegarty M (2004). Mechanical reasoning by mental simulation. Trends in Cognitive Sciences.

[CR19] Hegarty M, Steinhoff K (1997). Individual differences in use of diagrams as external memory in mechanical reasoning. Learning and Individual Differences.

[CR20] Hetherington JP, Warner A, Seymour RM (2006). Simplification and its consequences in biological modelling: Conclusions from a study of calcium oscillations in hepatocytes. Journal of the Royal Society, Interface.

[CR21] Hood L, Heath JR, Phelps ME, Lin B (2004). Systems biology and new technologies enable predictive and preventative medicine. Science.

[CR22] Humphreys P (2004). Extending ourselves: Computational science, empiricism, and scientific method.

[CR23] Humphreys P (2009). The philosophical novelty of computer simulation methods. Synthese.

[CR61] Hutchins, E. (1995). *Cognition in the wild*. MIT Press.

[CR24] Jones N, Wolkenhauer O (2012). Diagrams as locality aids for explanation and model construction in cell biology. Biology and Philosophy.

[CR25] Kitano H (2002). Looking beyond the details: A Rise in system-oriented approaches in genetics and molecular biology. Current Genetics.

[CR26] Lenhard J (2006). Surprised by a nanowire: Simulation, control, and understanding. Philosophy of Science.

[CR27] Lenhard J (2007). Computer simulation: The cooperation between experimenting and modeling. Philosophy of Science.

[CR28] Levins R (1966). The strategy of model building in population biology. American Scientist.

[CR29] MacLeod M, Nersessian NJ (2013). Building simulations from the ground-up: Modeling and theory in systems biology. Philosophy of Science.

[CR30] MacLeod M, Nersessian NJ (2013). Coupling Simulation and experiment: The bimodal strategy in integrative systems biology. Studies in History and Philosophy of Biological and Biomedical Sciences.

[CR31] MacLeod M, Nersessian NJ (2013). The creative industry of systems biology. Mind & Society.

[CR32] MacLeod M, Nersessian NJ (2014). Strategies for coordinating experimentation and modeling in integrative systems biology. Journal of Experimental Zoology Part B: Molecular and Developmental Evolution.

[CR33] Nersessian NJ (1984). Faraday to Einstein: Constructing meaning in scientific theories.

[CR34] Nersessian NJ, Giere R (1992). How do scientists think? Capturing the dynamics of conceptual change in science. Minnesota studies in the philosophy of science.

[CR35] Nersessian NJ, Carruthers P, Stich S, Siegal M (2002). The cognitive basis of model-based reasoning in science. The cognitive basis of science.

[CR37] Nersessian NJ (2008). Creating scientific concepts.

[CR38] Nersessian NJ (2009). How do engineering scientists think? Model-based simulation in biomedical engineering research laboratories. Topics in Cognitive Science.

[CR39] Nersessian NJ, MacLeod M, Mangani L, Bertolotti T (2017). Models and simulations. The Springer handbook of model-based science.

[CR40] Nersessian NJ, Patton C, Meijers A (2009). Model-based reasoning in interdisciplinary engineering: Two case studies from biomedical engineering research laboratories. Philosophy of technology and engineering sciences.

[CR41] Noble D (2008). The music of life: Biology beyond genes.

[CR43] Osbeck L, Nersessian NJ (2006). The distribution of representation. The Journal for the Theory of Social Behaviour.

[CR44] Parker WS (2010). Predicting weather and climate: Uncertainty, ensembles and probability. Studies in History and Philosophy of Science Part B: Studies in History and Philosophy of Modern Physics.

[CR45] Roschelle JR, Greeno JG (1987). Mental models in expert physics reasoning.

[CR60] Schwartz DL, Black JB (1996). Shuttling between depictive models and abstract rules: Induction and fallback. Cognitive Science.

[CR48] Thagard P (1988). Computational philosophy of science.

[CR49] Voit EO (2000). Computational analysis of biochemical systems: A practical guide for biochemists and molecular biologists.

[CR50] Voit EO (2013). A first course in systems biology.

[CR51] Voit EO (2013). Boichemical systems theory: A review. ISRN Bioinformatics.

[CR53] Voit EO, Newstetter WC, Kemp ML (2012). A feel for systems. Molecular Systems Biology.

[CR54] Voit EO, Qi Z, Kikuchi S (2012). Mesoscopic models of neurotransmission as intermediates between disease simulators and tools for discovering design principles. Pharmacopsychiatry.

[CR59] Weisberg M (2006). Robustness analysis. Philosophy of Science.

[CR55] Westerhoff HV, Kell DB, Boogerd F, Bruggeman FJ, Hofmeyer J-HS, Westerhoff HV (2007). The methodologies of systems biology. Systems biology: Philosophical foundations.

[CR58] Wimsatt, W. C. (2007). *Re-engineering philosophy for limited beings: Piecewise approximations to reality*. Harvard University Press.

[CR56] Winsberg E (2009). Computer simulation and the philosophy of science. Philosophy Compass.

[CR57] Winsberg E (2010). Science in the age of computer simulation.

